# Chronic disease stigma, skepticism of the health system, and socio-economic fragility: Qualitative assessment of factors impacting receptiveness to group medical visits and microfinance for non-communicable disease care in rural Kenya

**DOI:** 10.1371/journal.pone.0248496

**Published:** 2021-06-07

**Authors:** Rae Dong, Claudia Leung, Mackenzie N. Naert, Violet Naanyu, Peninah Kiptoo, Winnie Matelong, Esther Matini, Vitalis Orango, Gerald S. Bloomfield, David Edelman, Valentin Fuster, Simon Manyara, Diana Menya, Sonak D. Pastakia, Tom Valente, Jemima Kamano, Carol R. Horowitz, Rajesh Vedanthan

**Affiliations:** 1 Icahn School of Medicine at Mount Sinai, New York, NY, United States of America; 2 Duke University Medical Center, Durham, North Carolina, United States of America; 3 School of Arts and Social Sciences, Moi University, Eldoret, Kenya; 4 Academic Model Providing Access to Healthcare, Eldoret, Kenya; 5 Duke University School of Medicine, Durham, North Carolina, United States of America; 6 School of Public Health, Moi University, Eldoret, Kenya; 7 Center for Health Equity and Innovation, Purdue University College of Pharmacy, West Lafayette, Indiana, United States of America; 8 Keck School of Medicine of USC, Los Angeles, California, United States of America; 9 College of Health Sciences, Moi University, Eldoret, Kenya; 10 NYU Grossman School of Medicine, New York, NY, United States of America; University of British Columbia, CANADA

## Abstract

**Background:**

Non-communicable diseases (NCDs) are the leading cause of mortality in the world, and innovative approaches to NCD care delivery are being actively developed and evaluated. Combining the group-based experience of microfinance and group medical visits is a novel approach to NCD care delivery. However, the contextual factors, facilitators, and barriers impacting wide-scale implementation of these approaches within a low- and middle-income country setting are not well known.

**Methods:**

Two types of qualitative group discussion were conducted: 1) mabaraza (singular, baraza), a traditional East African community gathering used to discuss and exchange information in large group settings; and 2) focus group discussions (FGDs) among rural clinicians, community health workers, microfinance group members, and patients with NCDs. Trained research staff members led the discussions using structured question guides. Content analysis was performed with NVivo using deductive and inductive codes that were then grouped into themes.

**Results:**

We conducted 5 mabaraza and 16 FGDs. A total of 205 individuals (113 men and 92 women) participated in the mabaraza, while 162 individuals (57 men and 105 women) participated in the FGDs. In the context of poverty and previous experiences with the health system, participants described challenges to NCD care across three themes: 1) stigma of chronic disease, 2) earned skepticism of the health system, and 3) socio-economic fragility. However, they also outlined windows of opportunity and facilitators of group medical visits and microfinance to address those challenges.

**Discussion:**

Our qualitative study revealed actionable factors that could impact the success of implementation of group medical visits and microfinance initiatives for NCD care. While several challenges were highlighted, participants also described opportunities to address and mitigate the impact of these factors. We anticipate that our approach and analysis provides new insights and methodological techniques that will be relevant to other low-resource settings worldwide.

## Introduction

Non-communicable diseases (NCDs) are the leading cause of mortality in the world, with 80% of this burden occurring in low- and middle-income countries (LMICs) [1]. Innovative approaches to NCD care delivery are being actively developed and evaluated. In particular, there is increasing recognition that social determinants of health need to be incorporated into care delivery, in order to simultaneously address socio-economic as well as health issues [2, 3].

One potentially promising approach includes microfinance (MF) initiatives, which are financial services targeted at individuals, groups of individuals, or small businesses, to provide individuals with access to saving mechanisms and loan opportunities [4–7]. MF activities have been shown to reduce poverty and improve health outcomes [8]. Another innovative care delivery approach is the group medical visit (GMV), which is a clinical encounter involving a group of patients, and has been shown to increase the efficiency of care delivery, quality of care, enhance social support, and encourage self-efficacy [9, 10]. Combining the group-based experience of MF with a GMV is a novel approach to NCD care delivery that has the synergistic potential to improve health care access and quality, increase the strength of social networks among group members, and improve clinician-patient trust, in addition to other social determinants of health. We have previously reported beneficial impact from integrated GMV and MF in a small pilot study in western Kenya [11]. However, the contextual factors, facilitators, and barriers impacting wide-scale implementation of these approaches within an LMIC setting are not well studied.

The Bridging Income Generation with GrouP Integrated Care (BIGPIC) study in western Kenya is evaluating the impact of MF and GMVs on cardiovascular risk reduction among individuals with and at increased risk of diabetes [12]. The formative phase of this study aimed to identify the contextual factors, facilitators, and barriers that may impact the success of this approach. In this paper, we report the results of that pre-implementation formative inquiry.

## Methods

### Setting

The Academic Model Providing Access to Healthcare (AMPATH) is a partnership between Moi University College of Health Sciences in western Kenya, Moi Teaching and Referral Hospital, and a consortium of North American academic medical centers [13]. AMPATH established a system of care delivery for HIV patients in 2001. Subsequently, in response to the growing burden of chronic disease (particularly diabetes and hypertension) within the population [14], expanded its clinical scope to include primary health care and chronic disease management serving a catchment area of over 4 million people [15]. The chronic disease management program primarily provides health facility-based care for patients with diabetes and hypertension.

Ethics approval was obtained from NYU Grossman School of Medicine Institutional Review Board, Icahn School of Medicine Institutional Review Board, and Moi University Institutional Research and Ethics Committee. All participants provided verbal informed consent prior to participating in the study.

### Participants and procedures

For this qualitative study, community members were invited to join mabaraza (singular, baraza), a traditional East African community gathering used as a form of participatory research to discuss and exchange information regarding a variety of topics and issues in a large heterogeneous group setting [16]. We worked with AMPATH leadership and local community leaders to organize “health mabaraza” in each local community, with ~40 participants each. For each baraza, we issued invitations to the local leadership with a description of the topic, and the general community was invited.

To complement the mabaraza, we conducted focus group discussions (FGDs) of 10–15 participants each, targeting specific groups with shared characteristics. We formed the FGDs by purposive sampling to achieve diversity of age, gender, occupation, and distance from the nearest health facility. Kenyans of all race/ethnic backgrounds were included. We recruited participants from three different groups: 1) individuals with diabetes or hypertension; 2) microfinance group members; and 3) rural health workers.

Community entry and community engagement, in partnership with community leaders, was conducted in each of the communities where discussions were held prior to the initiation of the qualitative sessions. All qualitative sessions occurred from August to October 2015, and took place at publicly accessible gathering sites within the community. No one was present at sessions beyond the participants and research staff. Participants were made aware that group facilitators were part of the BIGPIC research team; beyond this, no personal characteristics about the facilitators were shared with participants. Participants engaged in one session each, without repeat participation. Each session lasted about 60 minutes, and sessions were concluded once data saturation was felt to have been achieved.

Structured question guides were developed to include content related to experience of chronic disease care, facilitators and barriers to GMVs, the role of microfinance in promoting health, and factors that might impact joining and remaining in groups. These question guides were pilot-tested on community members, patients, and clinicians prior to being used in the qualitative sessions. Three female research staff members (PK, WM, EM) were trained in group facilitation using standardized materials, and trained in use of the guides. Fluent in the local languages, they led the discussions in English and/or Kiswahili, as was appropriate for the participants. Beyond this initial structure, the discussions were allowed to deviate as additional relevant issues emerged. Facilitators took care to maintain a neutral role and maintain an open and balanced flow of ideas from all attendees.

Sessions were audio-recorded, transcribed, and translated to English. Field notes were also captured by the moderator at the time of the each session. Content analysis was performed with NVivo using deductive and inductive codes that were then grouped into themes. A kappa score of > 0.90 was established as the threshold to ensure inter-rater reliability among three independent coders (RD, CL, MN). Several thematic analysis working group meetings were held among research team members to examine and discuss common topics, ideas, and patterns, which were aggregated into three over-arching themes: stigma of chronic disease, skepticism of the health system, and socioeconomic fragility (Table 1). Participants were not re-interviewed for feedback on the coding scheme or thematic analysis.

**Table 1:** Summary of the themes and sub-themes that arose from the content analysis.

**Chronic disease stigma** o Fear o Motivation o Respect o HIV Care (as a point of comparison) o Social support o Instrumental support o Trust and sharing o Cohesion & Belonging	**Health system skepticism** o Quality of care o Respect o Perceptions of doctors’ motivations	**Socio-economic fragility** o Infrastructure o Availability o Adherence o Cost o Time o Medications

## Results

In total, 21 qualitative sessions (5 mabaraza and 16 FGDs) were conducted in 11 distinct geographic regions in western Kenya. A total of 205 individuals (113 men and 92 women) participated in the mabaraza, while 162 individuals (57 men and 105 women) participated in the FGDs.

### Context

In general, each category of participant had heard of microfinance. In contrast, none of the participants had previously heard of GMV with the exception of a CHW who facilitated the creation of a group of patients living with HIV. Clinicians, microfinance group members and patients in general thought that women would be more interested in GMV and MF than men, particularly because more women in this region tend to participate in microfinance. However, participants also felt men would be interested in joining with adequate education and information.

**Fig 1:**
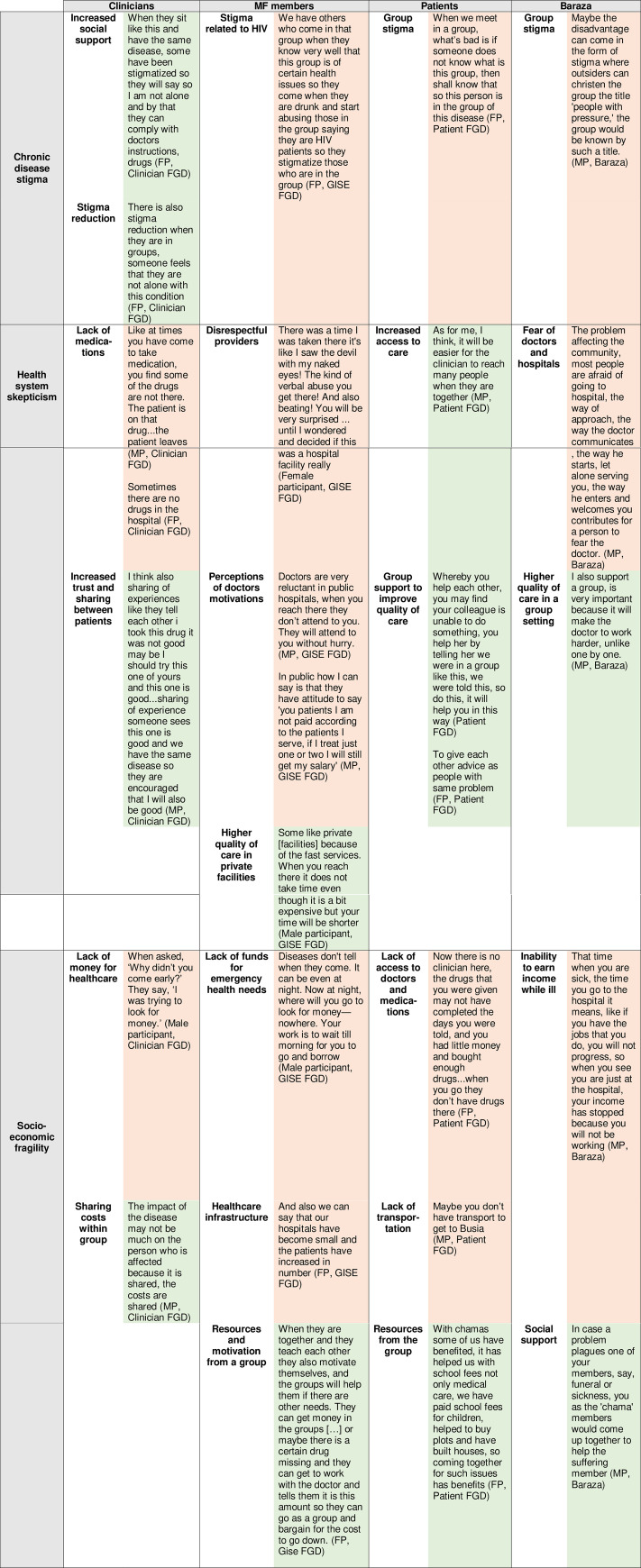
Matrix analysis of themes, sub-themes, and participant category. Quotations representing potential facilitators of GMV/MF are shaded in green while quotations expressing potential barriers are shaded in red. FGD = Focus Group Discussion; FP = Female Participant; GISE = Group Integrated Savings for Empowerment (Microfinance Group); GMV = Group Medical Visit; MF = Microfinance; MP = Male Participant.

Clinicians, microfinance group members, patients, and baraza participants noted several barriers to chronic disease care across three overall themes of chronic disease stigma, health system skepticism, and socioeconomic fragility. However, they noted that there were potential windows of opportunity and facilitators of a combined GMV-MF approach that could potentially navigate the challenges (Fig 1).

### Stigma of chronic disease

Participants noted that, specific to NCDs, there was the potential for stigma and being considered a distinct ‘other,’ characterized by undesirable status or negative stereotypes. With respect to group-based MF or GMV, there was concern among patients, baraza participants and microfinance group members that membership in the group would lead to being labeled as “sick” and potentially “inferior.” Conversely, some clinicians expressed optimism that participation in MF or GMV could increase a sense of “belonging,” acceptance, and social cohesion, which could counter the potential for negative stigma. Clinicians also expressed that decreased stigma could lead to increased adherence with medications and medical treatment.

### Skepticism of the health system

Skepticism of the health system was described regarding both the overall quality of care provided, as well as trust in clinical providers. Much of this skepticism was grounded in patients having had previous negative experiences with the health system and clinicians. Patients and microfinance members reported experiencing a lack of respect, verbal abuse, and not getting adequate or comprehensive services. Clinicians also expressed skepticism in the health system with respect to inadequate supply of medications and understaffing in hospitals that impacted their ability to take care of patients.

There were some notable differences in previous experiences in the public vs. private sector, but neither sector was free from criticism or concern. For instance, patients and microfinance members reported that in the private sector, doctors’ actions are felt to be driven by money and commercial interests, and they might not have patients’ best interests at heart. In contrast, public-sector health providers who are paid a salary are not incentivized to provide services for the purposes of making more money. These providers were described as being “serious” and “more professional.” However, participants also reported the opposite experience, where private-sector providers were seen as providing higher quality care because they are incentivized to treat patients better in order to increase their income, in contrast to public doctors who are not necessarily incentivized to provide quality care in this way. Private sector health facilities were also viewed as being more efficient and clean, but more expensive than the public sector, which was described as being less expensive but of poor quality.

All categories of participant felt that GMV, in particular, and MF had the potential to lead to increased clinician engagement and accountability. Given that a group of patients would be together for a GMV, patients and community members felt that the clinician would be more responsive, more respectful, and more accountable. In addition, it was felt that MF and GMV would increase both social and instrumental support with respect to access to care. Specifically, the group-based format could serve as an avenue for advocacy and for increasing the confidence to advocate on behalf of oneself and other group members.

### Socio-economic fragility

Study participants described a nearly all-encompassing sense of socioeconomic fragility that adversely impacted the entire care cascade, from being screened to seeking care to affording medications to completing follow-up visits. For example, lack of access to medicines due to cost was considered a major barrier to experiencing positive health outcomes. In addition, poor health and unplanned illness were felt to further exacerbate an individual’s and family’s economic strain due to the cost of medical care, as well as lost wages.

Socio-economic fragility was felt to worsen the impact of previously described stigma and health system skepticism. Participants reported that challenges with health care access due to affordability would adversely affect both real and perceived quality of care received by patients. In a negatively reinforcing cycle, the poorer quality of care would exacerbate health system skepticism, leading to lower healthcare utilization, delayed care-seeking, and lower adherence to medical advice, resulting in even worse health outcomes.

The combination of GMV and MF were felt to hold promise for addressing this socio-economic fragility. MF was felt to directly increase liquidity and purchasing capacity, and indirectly to improve overall income-earning potential. This could enable healthier behaviors such as improved diet, medication adherence, and ability to pay for medicines and medical services. In addition, GMV was felt to potentially increase social support, group cohesion, and a sense of belonging, thereby increasing motivation and capacity for economic and health improvement. MF in conjunction with GMV were felt to synergistically improve behavior change, medication adherence, retention in care, and increased health knowledge.

## Discussion

**Fig 2:**
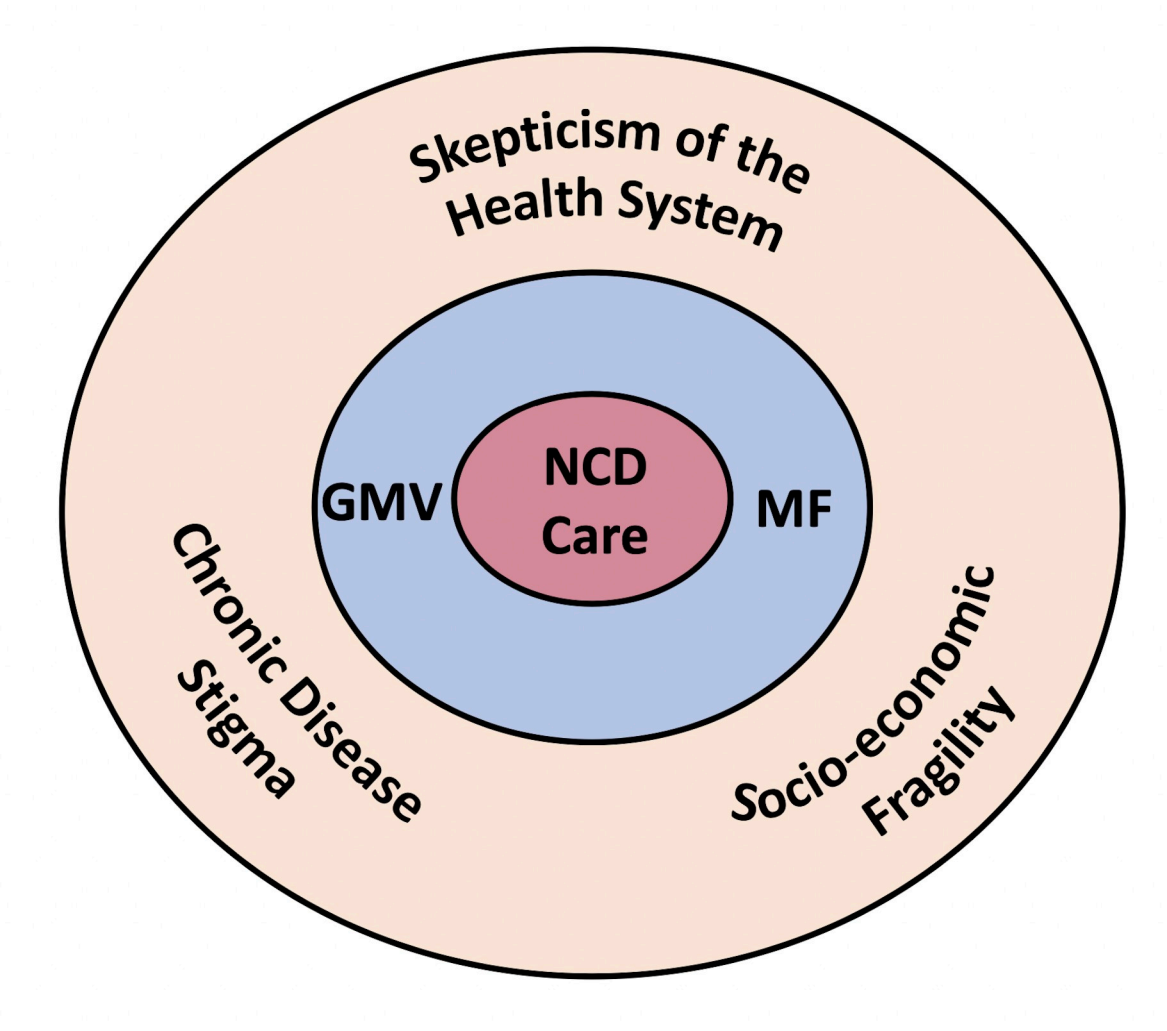
Conceptual representation of challenges to NCD care that can be mitigated by GMV and MF. Conceptual model.

In this qualitative study from western Kenya, we found that chronic disease stigma, skepticism of the health system, and socio-economic fragility were all factors that could impact the potential implementation success of GMV and MF for patients with diabetes and hypertension (Fig 2). Importantly, all three factors were reported as potential barriers for any NCD program and were based on historical experiences that did not necessarily include previous exposure to GMV or MF. Conversely, participants also felt that GMV and MF could potentially address and mitigate the impact of these dynamic factors. While our study population were generally familiar with MF, the GMV was a novel concept. Participants repeatedly expressed that they perceived value in belonging to a group, and that incorporating GMV into MF was a way of leveraging social networks that already existed in the form of MF groups to improve NCD care delivery.

Stigma has commonly been associated with infectious diseases such as HIV, and HIV-related stigma and discrimination have been well established as barriers to accessing HIV prevention, treatment, and support services [17]. Our group has previously reported that co-locating hypertension management in the same facility as HIV care can present challenges due to HIV-related stigma [18]. However, in the current study, participants described NCD-specific stigma that could act as a barrier to care. Others have reported that individuals with NCDs feel like they are blamed for their own illness by community members and health care workers [19]. In particular, individuals who anticipated greater stigma from health care workers have been found to be less likely to access health care due to the prior negative experiences [20]. Specific to the group-based GMV and MF activities proposed in this study, stigma may lead to fear of joining a patient group because being linked to the group may be associated with negative stereotyping, lower social status, and discrimination.

Stigma related to health care workers’ attitudes towards patients with NCDs may contribute to the health system skepticism described by participants, as described above. In addition, participants reported instances of verbal abuse and lack of being respectfully treated by health care staff. Perceived low quality of care has been corroborated by empirical data indicating poor quality of care in LMICs [21]. The adverse experiences described by our participants led to skepticism, lack of confidence, and lack of trust in the health system, which again has been widely reported in other parts of the world [22]. Unfortunately, mistrust in clinical providers can lead to lower adherence to medical advice and subsequent poor health outcomes [23, 24]. Skepticism of the health system has also been associated with lower health care utilization, lower rates of adoption of prevention interventions, and higher rates of unhealthy behaviors [25]. This self-perpetuating, negatively reinforcing cycle yields adverse outcomes for individuals, populations, and health systems [26]. Thus, it is imperative to break this cycle by improving quality of care, re-gaining trust of patients and community members, and disseminating these successes to the broader population.

Socio-economic fragility, in our population, appeared to exacerbate the potential negative sequelae of stigma and health care skepticism. Low socio-economic status is known to be associated with increased morbidity and mortality, although the mechanisms responsible for this are not fully established [27]. In Kenya specifically, it has been demonstrated that poorer households in rural areas are more likely to experience catastrophic out-of-pocket expenses, primarily related to payments for outpatient services [28]. At the societal level, socio-economic inequality is associated with disparities in NCD burden [29]. In our setting, all of the above dynamics appeared to be relevant. We have previously described substantial levels of material deprivation and lack of health insurance in western Kenya [30], thus lending support to care delivery models, such as BIGPIC, that incorporate social determinants of health into clinical care [11].

Participants in general felt that, despite the barriers presented by stigma, skepticism, and socio-economic fragility, the combination of GMV and MF could potentially address those barriers and be successful despite those factors. In particular, the anticipated social network benefit of GMV could synergistically interact with the economic benefit of MF to further enhance both health and financial outcomes, beyond what might be possible with each component individually. Given that this qualitative inquiry was the formative component of a larger implementation research trial [12], we have been vigilant to incorporate the findings from this inquiry into the design of the BIGPIC intervention using a stakeholder-based, human-centered design process [31]. At the same time, we recognize that our planned intervention will not be able to fully solve all of the potential issues, such as poverty and lack of health insurance. We are therefore heartened by the rollout and scale-up of universal health coverage programs in Kenya and other LMICs, which will provide much-needed financial risk protection for these populations [32].

We acknowledge the following limitations of our study. First, while we attempted to involve multiple stakeholder groups, it is likely that not all stakeholder perspectives were fully represented in this qualitative study. The overall BIGPIC project has other components that involve stakeholder engagement, such as the human-centered design process, in order to secure broader and deeper stakeholder participation throughout the implementation research project. Second, we recognize the potential for limited generalizability, since we recruited participants from specific geographic areas in western Kenya. Several of the salient themes, however, are consistent with findings from literature arising from other geographies, as discussed above, thus indicating that elements are indeed relevant for similar settings worldwide. Third, we did not record individual-level demographic information for the quotations and transcript. However, we view the themes as arising from a collective discussion, not necessarily from any one specific individual.

## Conclusions

NCDs are the leading cause of mortality in the world, and there is increasing recognition of the need to simultaneously address socio-economic as well as health issues in NCD management. Qualitative inquiry, as we have conducted in this study, is helpful to reveal and illuminate factors that may positively and negatively impact implementation success. The factors highlighted in our analysis—chronic disease stigma, skepticism of the health system, and socio-economic fragility—have clearly informed the design, development, and implementation of our group-based GMV and MF strategies for optimizing NCD management in western Kenya. We anticipate that our approach and analysis provides new insights and methodological techniques that may be relevant to other low-resource settings worldwide.

## Supporting information

S1 Checklist COREQ (COnsolidated criteria for REporting Qualitative research) checklist. (PDF)

S1 File (DOCX)
