# Supplementary material for: Chronic disease stigma, skepticism of the health system, and socio-economic fragility: Qualitative assessment of factors impacting receptiveness to group medical visits and microfinance for non-communicable disease care in rural Kenya
Source: PLoS One. 2021 Jun 7;16(6):e0248496. doi: 10.1371/journal.pone.0248496 (PMC8183981; doi:10.1371/journal.pone.0248496)
Supplement: S1 File — (DOCX) [file pone.0248496.s002.docx]

**Mabaraza Guide**

**STUDY:** **Bridging Income Generation with Group Integrated Care**

**Cover Sheet**

**Baraza moderator: ____________________________________________**

**Baraza recorder: ______________________________________________**

**Date: ____/____/2015**

**Time: ______________________________________________**

**Community Units: ___________________________________**

**Location/Venue: ____________________________________**

**Consent (no or yes) from participants: __________________**

**Baraza Completion Check List:**

**( ) Participant verbal informed consent read to the participants?**

**( ) Study cover sheet attached to field & discussion notes?**

**********************************************************

**Baraza Guide**

Background: AMPATH is working to implement an integrated microfinance-group medical visit program for diabetes and hypertension patients. We wish to understand how to offer services that would be most useful for your community members.

Opening Remarks

Hello. My name is ______XXX_________ and I will help guide our discussion today. Thank you for welcoming us to your community. ___XXX___________ is also present today to help record our conversations. This helps me to capture what you say, not what I think you said. Your opinions and experiences are very important to us. I want to hear what you have to say because it will help us to understand how to implement an integrated microfinance-group medical visit program for diabetes and hypertension patients. You can help us do a better job by giving us your honest opinions.

In this discussion, I consider you the teacher. I want your honest opinions and ideas. You should feel free to say whatever you think and feel. You are welcome to say as much or as little as you want. I am eager to hear what you have to say. Do you have any questions about what I have said so far?

Baraza Rules

1. There are no right or wrong answers. We expect that you will have differing points of view. Please feel free to share your point of view even if it differs from what others have said.
2. We are recording this session as we discussed, because we do not want to miss any of your comments. No names will be included in any reports. Your comments are confidential. Please speak loudly for I want to be accurate in capturing what you say.
3. Don’t feel like you have to respond all the time. But if you want to follow up on something that someone has said, you want to agree, or disagree, or give an example, feel free to do that.
4. I am here to ask questions, listen, and make sure everyone has a chance to share. We’re interested in hearing from each of you. So if you are talking a lot, I may ask you to give others a chance.. We just want to make sure all of you have a chance to share your ideas.
5. If you have a cell phone, please put it on quiet mode.

Introductory Questions

1. What are some of the health conditions that you and your community are concerned about?
2. How well do you feel that these conditions are managed by the health care system?
   - Probes:
     - Private sector
     - Public sector/facilities

Traditional healers

Key Questions

*Social network*

1. Who do people in your community talk to most frequently when they have an important issue (including non-health related)?
2. Who do community members go to for advice about health matters?
3. What source of information do community members trust most when it comes to health? (examples: radio, television, magazines, word of mouth)
4. Is there a sense of brotherhood (‘undugu’? ‘Ujamaa’? in your community? What are the benefits of this? Please explain your answer.

*Transition*

1. What has been the previous experience of patients receiving care at health facilities for chronic diseases like diabetes and hypertension?
   1. ***Probe for location:***
      1. Private sector
      2. Public sector/facilities
      3. Traditional healers
   2. ***Probe for type of experience***
      1. *Cost*
      2. *Cleanliness*
      3. *Quality*
      4. *Respect*
      5. *Doctor patient communication*
      6. *Hours / availability*
2. What are the biggest challenges you or your community members face when trying to get medical care for chronic diseases like diabetes and hypertension?

*Group medical visit*

1. Have you ever heard of a group medical visit?
   1. ***Note to moderator****: Provide an explanation here to all participants:*

**A group medical visit is where a group of patients come together for a joint clinic appointment. The group would come together to talk about a health topic (such as – diabetes or blood pressure). Then, each person would get their blood pressure, and blood sugar tested and meet with the clinician individually to discuss the test results and what needs to be done.**

1. Have any of you been part of group medical visit? If so, what did you think was good or bad about it?
2. What do people in your community think are the benefits of a group medical visit as compared with individual appointments (seeing the clinician one-on-one)?
   1. ***Note to the moderator:*** Some examples may include:
      1. A sense of camaraderie with other group members
      2. A sense of trust between group members
      3. Being more motivated by group members
      4. Provider may be more engaged with the group
      5. The provider may have more time to spend with the group
      6. Learning things from other people’s experiences or questions
3. What will people worry about when participating in a group medical visit?
   1. ***Note to the moderator***: Some examples may include:
      1. Worry about confidentiality (about something I say)
      2. Worry about privacy (about receiving care in a group)
      3. The provider will not be able to adequately address my individual needs
      4. Provider will focus on things that are not important to me
4. What would prevent people/patients from participating in group medical visits?
   1. ***Note to the moderator***: Some examples may include:
      1. Not enough time to commit to program (because visits will take too much time)
      2. Not enough money
      3. Lack of trust in the provider
5. How interested would people/patients be in participating in a group medical visit?
6. How much would people/patients be willing to pay (more, less, or the same) for health services delivered in a group setting?
   1. Probe for diabetes – would you be willing to pay more, less, or the same for the below services delivered to your group compared with traveling to a clinic?
      1. Blood glucose testing
      2. Drug refill (including insulin and needles, if needed)
      3. Counseling and patient education
   2. Probe for hypertension – would you be willing to pay more, less, or the same for the below services delivered to your group compared with traveling to a clinic?
      1. Blood pressure screening
      2. Drug refill
      3. Counseling and patient education

*Microfinance*

1. How much is cost a problem for getting the health care that people need?
   1. ***Follow up:*** *Is healthcare a big expense for you or your family?*
2. How much do families in your community spend on healthcare as a proportion of monthly income?
   1. Probe: *How does it compare to other expenses such as food and housing?*
3. Have you heard about microfinance programs in the past?
   1. ***Note to moderator****: Provide an explanation here to all participants:*

**Microfinance programs (chamas) provide chance for a group of people to save money together and also lend money to each other.**

1. How many of you have had any experience with microfinance in the past? If yes, what did you think was good or bad about it?
2. How much interest do you think patients would have in participating in a microfinance program combined with group medical visits?

Follow up: How interested would men be as compared with women? Why?

Ending Questions

1. Is there anything that we should have talked about but have not yet discussed?

Conclusion and Wrap-up

1. Thank you for your time and participation. We very much appreciate your comments, discussion, and input. We plan to take into account everything that was said today as we continue to improve the services we offer to your community.
2. If you have any questions or wish to discuss any of these issues further, please contact Dr. Rajesh Vedanthan c/o Dr. Jemima Kamano

Moi University College of Health Sciences

Eldoret, Kenya

shoine.hoine@gmail.com

+254-53-2060727

**Focus Group Discussion (FGD) Guide**

**STUDY:** **Bridging Income Generation with Group Integrated Care**

**Cover Sheet**

**FGD moderator: ____________________________________________**

**FGD recorder: ______________________________________________**

**Category of FGD (tick): Young Males & Females _____**

**Older Males & Females _____**

**Date: ____/____/201_**

**Time: _____________________________________________**

**Community Unit: ___________________________________**

**Location/Venue: ____________________________________**

**Consent (no or yes) from participants: __________________**

**FGD Completion Check List:**

**( ) Participant verbal informed consent read to the participants?**

**( ) Study cover sheet attached to field & discussion notes?**

************************************************************

**Focus Group Discussion Guide**

**Patients**

Background: AMPATH is working to implement group medical visits with patient with diabetes and hypertension. We wish to better understand how to offer services that would be the most useful for you.

Opening Remarks

Hello. My name is ______XXX_________ and I will help guide our discussion today. Thank you for welcoming us to your community. ___XXX___________ is also present today to help record our conversations. This helps me to capture what you say, not what I think you said. Your opinions and experiences are very important to us. I want to hear what you have to say because it will help us to understand how to implement an integrated microfinance-group medical visit program for diabetes and hypertension patients. You can help us do a better job by giving us your honest opinions.

In this discussion, I consider you the teacher. I want your honest opinions and ideas. You should feel free to say whatever you think and feel. You are welcome to say as much or as little as you want. I am eager to hear what you have to say. Do you have any questions about what I have said so far?

1. .

Focus Group Rules

1. There are no right or wrong answers. We expect that you will have differing points of view. Please feel free to share your point of view even if it differs from what others have said.
2. We are recording this session as we discussed, because we do not want to miss any of your comments. No names will be included in any reports. Your comments are confidential. Please speak loudly for I want to be accurate in capturing what you say.
3. Don’t feel like you have to respond all the time. But if you want to follow up on something that someone has said, you want to agree, or disagree, or give an example, feel free to do that.
4. I am here to ask questions, listen, and make sure everyone has a chance to share. We’re interested in hearing from each of you. So if you are talking a lot, I may ask you to give others a chance. We just want to make sure all of you have a chance to share your ideas.
5. If you have a cell phone, please put it on quiet mode.

Key Questions

*Social network*

1. Who do people in your community talk to most frequently when you have an important issue (including non-health related)?
2. Who do community members go to for advice about health matters?
3. What source of information do community members trust most when it comes to health? (examples: radio, television, magazines, word of mouth)
4. Is there a sense of brotherhood (‘undugu’? ‘Ujamaa’? in your community? What are the benefits of this? Please explain your answer.

*Transition*

1. What has been the previous experience of patients receiving care at health facilities for chronic diseases like diabetes and hypertension?
   1. ***Probe for location:***
      1. Private sector
      2. Public sector/facilities
      3. Traditional healers
   2. ***Probe for type of experience***
      1. *Cost*
      2. *Cleanliness*
      3. *Quality*
      4. *Respect*
      5. *Doctor patient communication*
      6. *Hours / availability*
2. What are the biggest challenges you or your community members face when trying to get medical care for chronic diseases like diabetes and hypertension?

*Group medical visit*

1. Have you ever heard of a group medical visit?
   1. ***Note to moderator****: Provide an explanation here to all participants:*

**A group medical visit is where a group of patients come together for a joint clinic appointment. The group would come together to talk about a health topic (such as – diabetes or blood pressure). Then, each person would get their blood pressure, and blood sugar tested and meet with the clinician individually to discuss the test results and what needs to be done.**

1. Have any of you been part of group medical visit? If so, what did you think was good or bad about it?
2. What do people in your community think are the benefits of a group medical visit as compared with individual appointments (seeing the clinician one-on-one)?
   1. ***Note to the moderator:*** Some examples may include:
      1. A sense of camaraderie with other group members
      2. A sense of trust between group members
      3. Being more motivated by group members
      4. Provider may be more engaged with the group
      5. The provider may have more time to spend with the group
      6. Learning things from other people’s experiences or questions
3. What will people worry about when participating in a group medical visit?
   1. ***Note to the moderator***: Some examples may include:
      1. Concerns regarding confidentiality (about something I say)
      2. Concerns regarding privacy (about receiving care in a group)
      3. The provider will not be able to adequately address my individual needs
      4. Provider will focus on things that are not important to me
4. What would prevent people/patients from participating in group medical visits?
   1. ***Note to the moderator***: Some examples may include:
      1. Not enough time to commit to program (because visits will take too much time)
      2. Not enough money
      3. Lack of trust in the provider
5. How interested would people/patients be in participating in a group medical visit?
6. How much would people/patients be willing to pay (more, less, or the same) for health services delivered in a group setting (e.g. a package of health services for the entire group of enrollees, including regular screenings, outpatient care, inpatient care, etc)
   1. Probe for diabetes – would you be willing to pay more, less, or the same for the below services delivered to your group compared with traveling to a clinic?
      1. Blood glucose testing
      2. Drug refill (including insulin and needles, if needed)
      3. Counseling and patient education
   2. Probe for hypertension – would you be willing to pay more, less, or the same for the below services delivered to your group compared with traveling to a clinic?
      1. Blood pressure screening
      2. Drug refill
      3. Counseling and patient education

*Microfinance*

1. How much is cost a problem for getting the health care that people need?
   1. ***Follow up:*** *Is healthcare a big expense for you or your family?*
2. How much do families in your community spend on healthcare as a proportion of monthly income?
   1. ***Follow up:*** *How does it compare to other expenses such as food and housing*
3. Have you heard about microfinance programs in the past?
   1. ***Note to moderator****: Provide an explanation here for all participants.*

**Microfinance programs (chamas) provide chance for a group of people to save money together and also lend money to each other.**

1. How many of you have had any experience with microfinance in the past? If yes, what did you think was good or bad about it?
2. How much interest do you think patients would have in participating in a microfinance program combined with group medical visits?
   1. Follow up: How interested would men be as compared with women? Why?

Ending Questions

1. Is there anything that we should have talked about but have not yet discussed?

Demographic Information and select personal data

| **Indicate or insert your responses as necessary** | | | | | | | |
| --- | --- | --- | --- | --- | --- | --- | --- |
| 1. State your age (Years) |  | | | | | | |
| 2. Sex | Male | Female | | | | | |
| 3. Education | No formal education | Primary | | Secondary | | Beyond secondary | Special training e.g Trade. *Insert* |
| 4. What is your principle form of labour or role in the community | Farming | Business/  trade | | Teacher | | Health worker | Other/s *(Insert)* |
| 5. Do you hold any special role in your community? | Local administrative leader | Healer | | Local spiritual leader | | Leader in a defined group e.g Chama, Peer network | Other/s *(Insert)* |
| 6. How far do you live from the nearest health facility | <5KM | >5KM | | | | | |
| 7. What is your estimated monthly income? | <Kshs. 20,000 | 21-50,000 | 51-100,000 | | 101-150,000 | | >150,000 |
| 8. How much of your annual income is spent on healthcare? | <Kshs.20,000 | 21-50,000 | 51-100,000 | | 101-150,000 | | >150,000 |
| 9. How long have you been a patient with AMPATH? | <3 months | Between 3 – 6 months | Between 6 months - 1year | | Between 1-2 years | | >2 years |

Conclusion and Wrap-up

1. Thank you for your time and participation. We very much appreciate your comments, discussion, and input. We plan to take into account everything that was said today as we continue to improve the services we offer to your community.
2. If you have any questions or wish to discuss any of these issues further, please contact Dr. Rajesh Vedanthan c/o Dr. Jemima Kamano

Moi University College of Health Sciences

Eldoret, Kenya

shoine.hoine@gmail.com

+254-53-2060727

**Focus Group Discussion (FGD) Guide**

**STUDY:** **Bridging Income Generation with group Integrated Care**

**Cover Sheet**

**FGD moderator: ____________________________________________**

**FGD recorder: ______________________________________________**

**Category of FGD (tick): Young Males & Females _____**

**Older Males & Females _____**

**Date: ____/____/2015**

**Time: _____________________________________________**

**Community Unit: ___________________________________**

**Location/Venue: ____________________________________**

**Consent (no or yes) from participants: __________________**

**FGD Completion Check List:**

**( ) Participant verbal informed consent read to the participants?**

**( ) Study cover sheet attached to field & discussion notes?**

************************************************************

**Focus Group Discussion Guide**

**Microfinance group member**

Background: AMPATH is working to implement a microfinance program for community members. We wish to better understand how to offer services that would be the most useful for you.

Opening Remarks

Hello. My name is ______XXX_________ and I will help guide our discussion today. Thank you for welcoming us to your community. ___XXX___________ is also present today to help record our conversations. This helps me to capture what you say, not what I think you said. Your opinions and experiences are very important to us. I want to hear what you have to say because it will help us to understand how to implement an integrated microfinance-group medical visit program for diabetes and hypertension patients. You can help us do a better job by giving us your honest opinions.

In this discussion, I consider you the teacher. I want your honest opinions and ideas. You should feel free to say whatever you think and feel. You are welcome to say as much or as little as you want. I am eager to hear what you have to say. Do you have any questions about what I have said so far?

Focus Group Rules

1. There are no right or wrong answers. We expect that you will have differing points of view. Please feel free to share your point of view even if it differs from what others have said.
2. We are recording this session as we discussed, because we do not want to miss any of your comments. No names will be included in any reports. Your comments are confidential. Please speak loudly for I want to be accurate in capturing what you say.
3. Don’t feel like you have to respond to me all the time. But if you want to follow up on something that someone has said, you want to agree, or disagree, or give an example, feel free to do that.
4. I am here to ask questions, listen, and make sure everyone has a chance to share. We’re interested in hearing from each of you. So if you are talking a lot, I may ask you to give others a chance. We just want to make sure all of you have a chance to share your ideas.
5. If you have a cell phone, please put it on quiet mode.

Key Questions

*Social network*

1. Who do people in your community talk to most frequently when you have an important issue (including non-health related)?
2. Who do community members go to for advice about health matters?
3. What source of information do community members trust most when it comes to health? (examples: radio, television, magazines, word of mouth)
4. Is there a sense of brotherhood (‘undugu’? ‘Ujamaa’? in your community? What are the benefits of this? Please explain your answer.

*Transition*

1. What has been the experience of patients receiving care at health facilities for chronic diseases like diabetes and hypertension?
   1. ***Probe for location:***
      1. Private sector
      2. Public sector/facilities
      3. Traditional healers
   2. ***Probe for type of experience***
      1. *Cost*
      2. *Cleanliness*
      3. *Quality*
      4. *Respect*
      5. *Doctor patient communication*
      6. *Hours / availability*
2. What are the biggest challenges you or your community members face when trying to get medical care for chronic diseases like diabetes and hypertension?

*Group medical visit*

1. Have you ever heard of a group medical visit?
   1. ***Note to moderator****: Provide an explanation here to all participants:*

**A group medical visit is where a group of patients come together for a joint clinic appointment. The group would come together to talk about a health topic (such as – diabetes or blood pressure). Then, each person would get their blood pressure, and blood sugar tested and meet with the clinician individually to discuss the test results and what needs to be done.**

1. Have any of you been part of group medical visit? If so, what did you think was good or bad about it?
2. What do people in your community think are the benefits of a group medical visit as compared with individual appointments (seeing the clinician one-on-one)?
   1. ***Note to the moderator:*** Some examples may include:
      1. A sense of camaraderie with other group members
      2. A sense of trust between group members
      3. Being more motivated by group members
      4. Provider may be more engaged with the group
      5. The provider may have more time to spend with the group
3. What will people worry about when participating in a group medical visit?
   1. ***Note to the moderator***: Some examples may include:
      1. Concerns regarding confidentiality (about something I say)
      2. Concerns regarding privacy (about receiving care in a group)
      3. The provider will not be able to adequately address my individual needs
      4. The provider will focus on things that are not important to me
4. What would prevent people/patients from participating in group medical visits?
   1. ***Note to the moderator***: Some examples may include:
      1. Not enough time to commit to program (because visits will take too much time)
      2. Not enough money
      3. Lack of trust in the provider
5. How interested would people/patients be in participating in a group medical visit?
6. How much would people/patients be willing to pay (more, less, or the same) for health services delivered in a group setting (e.g. a package of health services for the entire group of enrollees, including regular screenings, outpatient care, inpatient care, etc)
   1. Probe for diabetes – would you be willing to pay more, less, or the same for the below services delivered to your group compared with traveling to a clinic?
      1. Blood glucose testing
      2. Drug refill (including insulin and needles, if needed)
      3. Counseling and patient education
   2. Probe for hypertension – would you be willing to pay more, less, or the same for the below services delivered to your group compared with traveling to a clinic?
      1. Blood pressure screening
      2. Drug refill
      3. Counseling and patient education

*Microfinance*

1. How much is cost a problem for getting the health care that people need?
   1. ***Follow up:*** *Is healthcare a big expense for you or your family?*
2. How much do families in your community spend on healthcare as a proportion of monthly income?
   1. ***Probe:*** *How does it compare to other expenses such as food and housing?*
3. How confident do families feel about their ability to earn enough income to access health care when they need it?
   1. ***If low to moderate confidence:*** How often do you delay or decide not to seek healthcare because it is too expensive?
4. Have you heard about microfinance programs in the past?
   1. ***Note to moderator****: Provide an explanation here to all participants*

**Microfinance programs (chamas) provide chance for a group of people to save money together and also lend money to each other.**

1. How many of you have had any experience with microfinance in the past? If yes, what did you think was good or bad about it?
2. How much interest do you think people would have in participating in a microfinance program combined with group medical visits?
   1. Follow up: How interested would men be as compared with women? Why?

Ending Questions

1. Is there anything that we should have talked about but have not yet discussed?

Demographic Information and select personal data

| **Indicate or insert your responses as necessary** | | | | | | | |
| --- | --- | --- | --- | --- | --- | --- | --- |
| 1. State your age (Years) |  | | | | | | |
| 2. Sex | Male | Female | | | | | |
| 3. Education | No formal education | Primary | | Secondary | | Beyond secondary | Special training e.g Trade. *Insert* |
| 4. What is your principle form of labour or role in the community | Farming | Business/  Trade | | Teacher | | Health worker | Other/s *(Insert)* |
| 5. Do you hold any special role in your community? | Local administrative leader | Healer | | Local spiritual leader | | Leader in a defined group e.g Chama, Peer network | Other/s *(Insert)* |
| 6. How far do you live from the nearest health facility | <5KM | >5KM | | | | | |
| 7. What is your estimated monthly income? | <Kshs. 20,000 | 21-50,000 | 51-100,000 | | 101-150,000 | | >150,000 |
| 8. How much of your annual income is spent on healthcare? | <Kshs.20,000 | 21-50,000 | 51-100,000 | | 101-150,000 | | >150,000 |

Conclusion and Wrap-up

1. Thank you for your time and participation. We very much appreciate your comments, discussion, and input. We plan to take into account everything that was said today as we continue to improve the services we offer to your community.
2. If you have any questions or wish to discuss any of these issues further, please contact Dr. Rajesh Vedanthan c/o Dr. Jemima Kamano

Moi University College of Health Sciences

Eldoret, Kenya

shoine.hoine@gmail.com

+254-53-2060727

**Focus Group Discussion (FGD) Guide**

**STUDY:** **Bridging Income Generation with group Integrated Care**

**Cover Sheet**

**FGD moderator: ____________________________________________**

**FGD recorder: ______________________________________________**

**Category of FGD (tick): Young Males & Females _____**

**Older Males & Females _____**

**Date: ____/____/2015**

**Time: _____________________________________________**

**Community Unit: ___________________________________**

**Location/Venue: ____________________________________**

**Consent (no or yes) from participants: __________________**

**FGD Completion Check List:**

**( ) Participant verbal informed consent read to the participants?**

**( ) Study cover sheet attached to field & discussion notes?**

************************************************************

**Focus Group Discussion Guide**

**Rural Clinician**

Background: AMPATH is working to implement a combined group medical visit and microfinance program for community members. We wish to better understand how to offer services that would be the most useful for patients and community members.

Opening Remarks

Hello. My name is ______XXX_________ and I will help guide our discussion today. Thank you for welcoming us to your community. ___XXX___________ is also present today to help record our conversations. This helps me to capture what you say, not what I think you said. Your opinions and experiences are very important to us. I want to hear what you have to say because it will help us to understand how to implement an integrated microfinance-group medical visit program for diabetes and hypertension patients. You can help us do a better job by giving us your honest opinions.

In this discussion, I consider you the teacher. I want your honest opinions and ideas. You should feel free to say whatever you think and feel. You are welcome to say as much or as little as you want. I am eager to hear what you have to say. Do you have any questions about what I have said so far?

Focus Group Rules

1. There are no right or wrong answers. We expect that you will have differing points of view. Please feel free to share your point of view even if it differs from what others have said.
2. We are recording this session as we discussed, because we do not want to miss any of your comments. No names will be included in any reports. Your comments are confidential. Please speak loudly for I want to be accurate in capturing what you say.
3. Don’t feel like you have to respond all of the time. But if you want to follow up on something that someone has said, you want to agree, or disagree, or give an example, feel free to do that.
4. I am here to ask questions, listen, and make sure everyone has a chance to share. We’re interested in hearing from each of you. So if you are talking a lot, I may ask you to give others a chance. We just want to make sure all of you have a chance to share your ideas.
5. If you have a cell phone, please put it on quiet mode.

Introductory Questions

1. What different types of patients do you treat?

Key Questions

*Social network*

1. To your knowledge, who do patients talk to most frequently when they have an important issue (including non-health care related)?
2. To your knowledge, who do patients go to for advice about health matters?
3. To your knowledge, what source of information do patients trust most when it comes to health? (examples: radio, television, magazines, word of mouth)
4. To your knowledge, is there a sense of brotherhood (‘undugu’? ‘Ujamaa’? in your community? What are the benefits of this? Please explain your answer.

Transition Questions

1. What has been your experience taking care of patients with chronic diseases like diabetes and hypertension?
   1. ***Probe for location:***
      1. Private sector
      2. Public sector/facilities
2. What are the biggest barriers your patients face when they try to get medical care for chronic diseases like diabetes and hypertension?
   1. Probe:
      1. *Cost*
      2. *Cleanliness*
      3. *Quality*
      4. *Respect*
      5. *Doctor patient communication*
      6. *Hours / availability*

*Group medical visit*

1. Have you ever heard of a group medical visit?
   1. ***Note to moderator****: Provide an explanation here to all participants*

**A group medical visit is where a group of patients come together for a joint clinic appointment. The group would come together to talk about a health topic (such as – diabetes or blood pressure). Then, each person would get their blood pressure, and blood sugar tested and meet with the clinician individually to discuss the test results and what needs to be done.**

1. Have any of you provided care in a group medical visit? If so, what did you think was good or bad about it?
2. What would be the benefits of providing care for chronic conditions such as diabetes and hypertension in a group medical visit?
3. ***Note to the moderator:*** Some examples may include:
   - 1. A sense of camaraderie with other group members
     2. A sense of trust between group members
     3. Being more motivated by group members
     4. Provider may be more engaged with the group
     5. The provider may have more time to spend with the group
     6. Learning things from other people’s experiences or questions
4. What would be the challenges to providing care for chronic conditions such as diabetes and hypertension care to a group of patients in a group medical visit?
   - ***Note to the moderator***: Some examples may include:
     - Concerns regarding confidentiality (about information the patient shares)
     - Concerns regarding privacy (about providing care in a group)
     - The provider will not be able to adequately address individual patient needs
     - The provider will need to cover information that is not relevant to everyone in the group
5. In your opinion, what would prevent patients from participating in a group medical visits?
   - ***Note to the moderator***: Some examples may include:
     - Not enough time to commit to program
     - Not enough money
     - Lack of trust in the provider
6. How interested would patients be in participating in a group medical visit?
7. How interested would clinicians be in providing group medical visits?
8. How do you think clinicians should be paid for health services delivered in a group setting (e.g. a package of health services for the entire group of enrollees, including regular screenings, outpatient care, inpatient care, etc)
   - ***Follow up:*** What is your level of interest in performance based bonuses based on patient outcomes?

*Microfinance*

1. How much is cost a problem for getting the health care that patients need?
   - ***Follow up:*** *Is healthcare a big expense for patients or their families?*
2. By your estimate, how much of your patients’ monthly income is usually spent on healthcare?
   - ***Probe***: *How does it compare to other expenses such as food and housing?*
3. Please explain your current understanding of microfinance.
   1. ***Note to moderator****: Provide an explanation here to all participants:*

**Microfinance programs (chamas) provide chance for a group of people to save money together and also lend money to each other.**

1. Have you heard about microfinance programs in the past?
2. Given our discussion of microfinance groups, how interested would your patients be in participating in a microfinance group?
3. How interested do you think patients would be in participating in a group that combines both microfinance and a group medical visit?
   - Follow up: How interested would men be as compared with women? Why?

Ending Questions

1. Is there anything that we should have talked about but have not yet discussed?

Demographic Information and select personal data

| **Indicate or insert your responses as necessary** | | | | | | | |
| --- | --- | --- | --- | --- | --- | --- | --- |
| 1. State your age (Years) |  | | | | | | |
| 2. Sex | Male | Female | | | | | |
| 3. Education | No formal education | Primary | | Secondary | | Beyond secondary | Special training e.g Trade. *Insert* |
| 4. What is your principle form of labour or role in the community | Farming | Business/  trade | | Teacher | | Health worker | Other/s *(Insert)* |
| 5. Do you hold any special role in your community? | Local administrative leader | Healer | | Local spiritual leader | | Leader in a defined group e.g Chama, Peer network | Other/s *(Insert)* |
| 6. How far do you live from the nearest health facility | <5KM | >5KM | | | | | |
| 7. What is your estimated monthly income? | <Kshs. 20,000 | 21-50,000 | 51-100,000 | | 101-150,000 | | >150,000 |
| 8. How much of your annual income is spent on healthcare? | <Kshs.20,000 | 21-50,000 | 51-100,000 | | 101-150,000 | | >150,000 |

Conclusion and Wrap-up

1. Thank you for your time and participation. We very much appreciate your comments, discussion, and input. We plan to take into account everything that was said today as we continue to improve the services we offer to your community.
2. If you have any questions or wish to discuss any of these issues further, please contact Dr. Rajesh Vedanthan c/o Dr. Jemima Kamano

Moi University College of Health Sciences

Eldoret, Kenya

shoine.hoine@gmail.com

+254-53-2060727
